# The complete mitochondrial genome of *Haustorioides koreanus* Jo, 1988 (Crustacea: Amphipoda: Dogielinotidae)

**DOI:** 10.1080/23802359.2019.1710600

**Published:** 2020-01-14

**Authors:** Sang-Hwa Lee, Sang-Hui Lee, Byung-Jin Lim, Jinwook Back, Eunchong Sin, Myung-Hwa Shin

**Affiliations:** National Marine Biodiversity Institute of Korea, Seocheon, Republic of Korea

**Keywords:** *Haustorioides koreanus*, complete mitogenome, Dogielinotidae, Amphipoda

## Abstract

The mitochondrial genome of a dogielinotid amphipod, *Haustorioides koreanus*, was completely sequenced for the first time. The total mitogenome length of *H*. *koreanus* was 14,839 bp with 13 protein-coding genes, two ribosomal RNA genes, and 22 transfer RNA genes. The phylogenetic tree confirmed that *H. koreanus* belongs to the families Hyalellidae in the same clade and to the suborder Senticaudata within Amphipoda. This is the first record of the complete mitochondrial genome sequence of the family Dogielinotidae.

The genus *Haustorioides* Oldevig 1958, one of the sand-burrowing amphipods, is a member of the family Dogielinotidae (Oldevig 1958). The species of the genus *Haustorioides* are specialized fossorial members, and have been endemic to the North Pacific region (Bousfield 1970; Bousfield and Tzvetkova 1982; Jo 1988). Only seven species have been recorded to date; *H. gurjanovae* Bousfield and Tzvetkova 1982 (from North American Pacific), *H. indivisus* Jo 1988 (from Korea), *H. koreanus* Jo 1988 (from Korea), *H. latipalpus* Jo 1988 (from Korea), *H. magnus* Bousfield and Tzvetkova 1982 (from the North American Pacific), *H. munsterhjelmi* Oldevig 1958 (from Sachaline, Russia), and *H. nesogenes* Jo 1988 (from Korea). The species *H. koreanus* is the most common dogielinotid amphipod on intertidal sandy beaches along the coast of Korea. In this study, we provide the complete mitogenome sequences of *H. koreanus* Jo 1988.

The specimen was collected from the intertidal beach in the Yellow Sea, Byeonsan-myeon, Buan-gun, Korea (35°34′55.86′′N, 126°30′22.45′′E), 25 March 2019. A voucher specimen was deposited at the National Marine Biodiversity Institute of Korea (MABIK CR00246522). The total genomic DNA was extracted from its legs by the method of Asahida et al. (1996). The complete mitochondrial genome sequence was amplified by conducting two independent and overlapping PCR runs with forward and reverse primers designed in this study.

The complete mitogenome of *H. koreanus* was 14,839 bp in length (GenBank accession no. MN840593). It encodes 37 genes, 13 protein-coding genes (PCGs), two ribosomal RNA genes (rRNAs), and 22 transfer RNA genes (tRNAs) and with one non-coding regions of 677 bp. The base composition of the mitogenome was 30.4% for A, 17.6% for C, 19.2% for G, and 32.8% for T. There were four types of PCGs start codons, ATA (atp6, cox2 and nad2), ATT (atp8, cox1, and nad5), ATG (cox3, cytb, nad3–4, nad4L, and nad6), and TTG (nad1). The stop codons contain TAA (atp6, atp8, cox2–3, cytb, nad3–4, nad4L, and nad6) and TAG (nad1 and nad4). Also, the nucleotide ‘T’ at the end of some PCGs (cox1, nad2, and nad5) was used to avoid duplication between the genes.

To confirm the phylogenetic position of *H. koreanus*, other 10 species belonging to 8 families in the suborder Senticaudata, based on the mitogenome sequences available in GenBank, with a lysianassid species, *Onisimus nanseni*, as an outgroup (all the species were registered in the NCBI). A phylogenetic tree was reconstructed based on the concatenated data set of 13 PCGs using the maximum likelihood (ML) method with the GTR + G + I model with RAxMLGUI v. 1.5b1 (Silvestro and Michalak 2012), the bootstrap values were calculated from 1000 replicates.

As a result, *H. koreanus* was grouped with other three families, Hyalellidae, Hyalidae, and Talitridae, in one clade with high bootstrap value, belonging to the suborder Senticaudata within Amphipoda (Figure 1). This is the first recorded of the complete mitogenome sequence for a sand-burrowing amphipod *H. koreanus*, belonging to the family Dogielinotidae. It provides valuable genetic information for future phylogenetic and evolutionary studies on amphipods.

**Figure 1.**
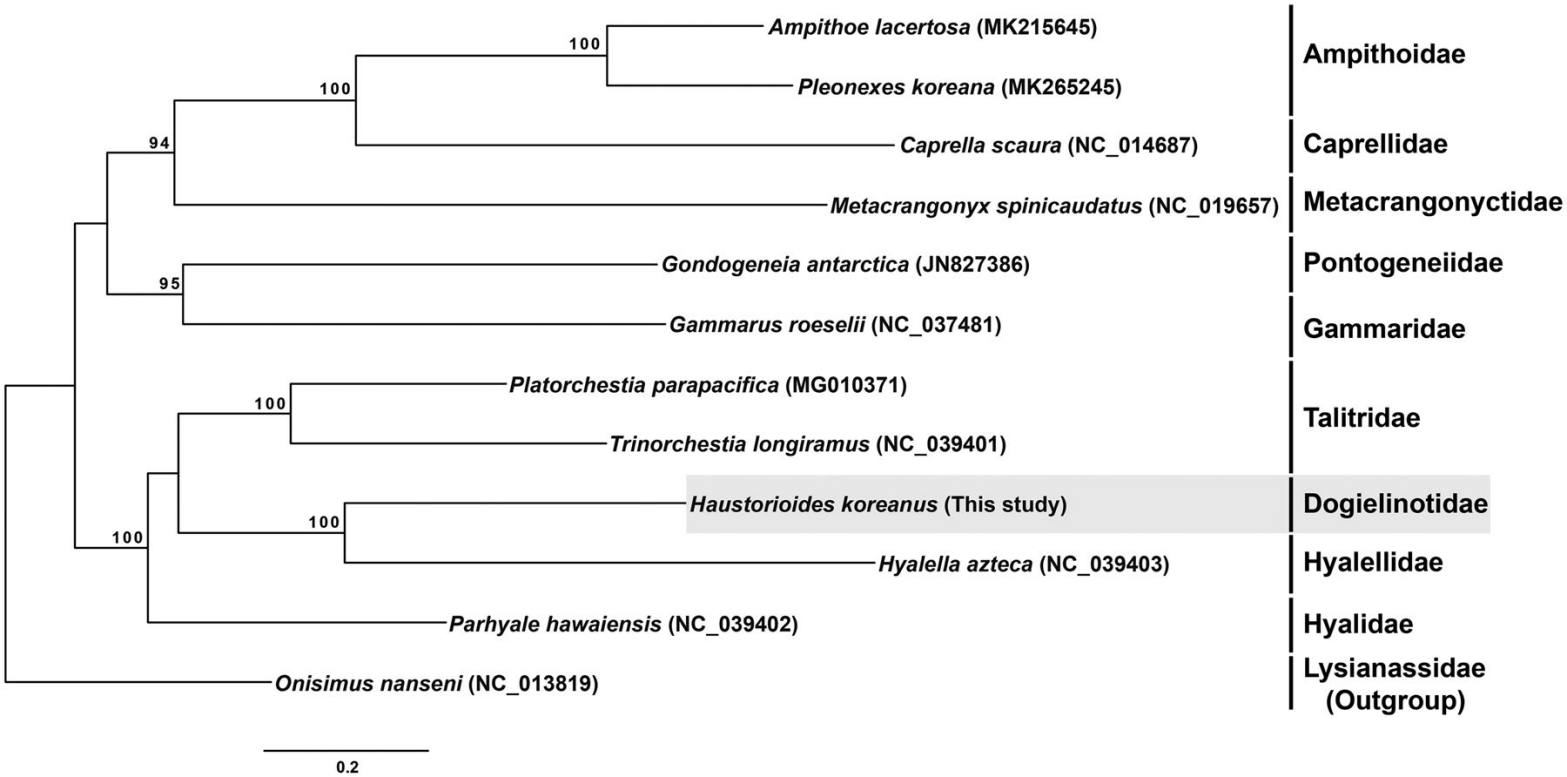
A phylogenetic tree built using the concatenated data set of 13 protein-coding genes by the maximum likelihood (ML) method. The tree was reconstructed based on 12 mitogenome sequences, including that of *Haustorioides koreanus* examined in this study. Bootstrap replicates were performed 1000 times. The sand-burrowing amphipod, *Haustorioides koreanus* is highlighted in gray.

## References

[CIT85697678] Asahida T, Kobayashi T, Saitoh K, Nakayama I. 1996. Tissue Preservation and Total DNA Extraction form Fish Stored at Ambient Temperature Using Buffers Containing High Concentration of Urea. Fisheries Science. 62(5):727–730.

[CIT0001] Bousfield EL. 1970. Adaptive radiation in sand-burrowing amphipod crustaceans. Chesapeake Sci. 11(3):143–154.

[CIT0002] Bousfield E, Tzvetkova N. 1982. Studies on Dogielinotidae (Amphipoda, Talitroidea) from the shallow waters of the North Pacific region. Explorations Fauna Seas. 29:76–94.

[CIT0003] Jo YW. 1988. Taxonomic studies on Dogielinotidae (Crustacea-Amphipoda) from the Korean Coast. BTD. 58(1):25–46.

[CIT0004] Oldevig H. 1958. On a new aberrant Talitrid from the Island of Sachalin. Arch Zool. 11:343–347.

[CIT0005] Silvestro D, Michalak I. 2012. raxmlGUI: a graphical front-end for RAxML. Org Divers Evol. 12:335–337.

